# The Implications for Cells of the Lipid Switches Driven by Protein–Membrane Interactions and the Development of Membrane Lipid Therapy

**DOI:** 10.3390/ijms21072322

**Published:** 2020-03-27

**Authors:** Manuel Torres, Catalina Ana Rosselló, Paula Fernández-García, Victoria Lladó, Or Kakhlon, Pablo Vicente Escribá

**Affiliations:** 1 Laboratory of Molecular Cell Biomedicine, Department of Biology, University of the Balearic Islands, Ctra. de Valldemossa km 7.5, E-07122 Palma, Spain; manuel.torres@luib.es (M.T.); ca.rossello@laminarpharma.com (C.A.R.); p.fernandez@laminarpharma.com (P.F.-G.); victoria.llado@laminarpharma.com (V.L.); 2 Department of R&D, Laminar Pharmaceuticals SL. ParcBit, Ed. Naorte B, E-07121 Palma, Spain; 3 Department of Neurology, Hadassah-Hebrew University Medical Center, Ein Kerem, 91120 Jerusalem, Israel; ork@hadassah.org.il

**Keywords:** protein–membrane interactions, melitherapy, lipid bilayer, membrane lipid switch, peripheral amphitropic non-permanently bound membrane proteins

## Abstract

The cell membrane contains a variety of receptors that interact with signaling molecules. However, agonist–receptor interactions not always activate a signaling cascade. Amphitropic membrane proteins are required for signal propagation upon ligand-induced receptor activation. These proteins localize to the plasma membrane or internal compartments; however, they are only activated by ligand-receptor complexes when both come into physical contact in membranes. These interactions enable signal propagation. Thus, signals may not propagate into the cell if peripheral proteins do not co-localize with receptors even in the presence of messengers. As the translocation of an amphitropic protein greatly depends on the membrane’s lipid composition, regulation of the lipid bilayer emerges as a novel therapeutic strategy. Some of the signals controlled by proteins non-permanently bound to membranes produce dramatic changes in the cell’s physiology. Indeed, changes in membrane lipids induce translocation of dozens of peripheral signaling proteins from or to the plasma membrane, which controls how cells behave. We called these changes “lipid switches”, as they alter the cell’s status (e.g., proliferation, differentiation, death, etc.) in response to the modulation of membrane lipids. Indeed, this discovery enables therapeutic interventions that modify the bilayer’s lipids, an approach known as membrane-lipid therapy (MLT) or melitherapy.

## 1. Introduction

The fluid mosaic model of cell membranes [1] contemplates the incorporation of integral transmembrane into their structure and their mobility in the bilayer as well as the association of peripheral proteins. Later studies demonstrated that these proteins participate in transmembrane communication in response to signals from neurotransmitters, hormones, cytokines, growth factors, etc. [2,3]. These productive interactions activate intracellular signaling cascades in which second and subsequent messengers regulate the expression of the genes that control the cell’s physiology [4]. In addition to this short-term messaging, mid- and long-term cytosolic and nuclear responses, the latter mediated by the regulation of gene expression, can affect the cell’s behavior over several hours, days, and even weeks [5]. In this regard, several issues must be considered. First, the physical interaction between membrane receptors and the amphitropic membrane proteins (either directly or through adaptor or scaffolding proteins) is necessary for the transduction of most cell signals [6,7]. Second, this interaction may not only depend on the expression of these proteins but also on the presence of the peripheral proteins in the vicinity of the membrane receptor, which may be controlled by membrane lipids [8,9]. Third, these interactions and the signals they produce are responsible for the pathophysiological status of the cell, which may be influenced by external cues, genetic alterations, alterations in membrane lipids, etc. [10,11]. Fourth, changes in the membrane lipid composition can induce important changes in the cell that affect proliferation, differentiation, and/or cell death [12,13]. Last but not least, the regulation of membrane lipids controls the type and abundance of the proteins in membranes, an approach that can be used to treat several conditions, including cancer, Alzheimer’s disease (AD), cardiovascular diseases (CVDs), inflammation, etc. [14,15,16,17].

In this context, the ability of membranes to generate microdomains due to the non-homogeneous mixing of membrane lipids is critical [18,19]. A variety of microdomains have been described in which either lamellar-prone or non-lamellar-prone lipids organize into different ordered or disordered lipid platforms [8,20,21,22]. These membrane regions with varying size can be distinguished from their adjacent microdomains in terms of their lipid and protein composition, bilayer thickness, lateral surface pressure, acyl chain mobility, membrane morphology, etc. Microdomains with a high proportion of hexagonal (H_II_) phase-prone lipids, such as phosphatidylethanolamine (PE) or diacylglycerol (DAG), are critical in the recruitment of peripheral amphitropic signaling proteins and thus, for cell growth and differentiation [8,23]. Recent studies showed that the proportion of peripheral amphitropic signaling proteins in membranes or aqueous compartments depends on both the membrane’s lipid composition, and the amino acid sequence involved in protein–lipid interactions and co/post-translational lipid modification of proteins [9,24]. Moreover, alterations to the peripheral signaling proteins at membranes and in the cytosol have been associated with a variety of pathologies [25,26]. The ability to signal proteins to translocate from the plasma membrane to intracellular compartments is a fundamental means to regulate cell signaling. Indeed, the interactions of messengers with their membrane receptors are nullified if the conformational change induced is not propagated from the receptor to its transducer.

Protein–lipid interactions have a strong influence on the activity of cells, and, therefore, it is important to investigate these interactions in basic biomedical and clinical studies. A number of issues have to be considered, including how a protein’s structure defines its presence in membranes, their abundance in different membrane regions or microdomains or in the cytoplasm, and how protein structure regulates protein–lipid interactions. In addition, it is important to consider how the membrane’s structure influences protein–lipid interactions, and how the pathophysiological and pharmaceutical/nutraceutical regulation of membrane composition affects cell signaling. Studies with specific proteins and lipids will be addressed in the first part of this review to explain how these interactions occur. Subsequently, the coordinated effect of several proteins and lipids on general pathophysiological processes will be addressed through what we define as “lipid switches”.

## 2. How Protein Structure Influences Protein–Lipid Interactions

Peripheral amphitropic proteins can translocate from aqueous to membranous compartments and they display a variety of structural features that define their interactions with membranes. These proteins bear lipid or amino acid motifs that drive their interactions with specific lipid species or lipid structures. In these interactions, the structure of both elements is crucial to understand how they occur and how they can be modulated in relation to the cell’s physiology. We first address the structural elements of proteins that influence their binding to cell membranes.

Co-translational and post-translational lipid modifications in proteins are critical to influence protein–lipid interactions, fulfilling roles above and beyond the mere binding of the protein to the lipid bilayer [27]. These modifications contribute to the peripheral and transmembrane localization of proteins at/in specific organelle membranes and membrane regions or microdomains [9,21,23,28,29,30]. In addition, certain amino acid domains in proteins that drive their binding to lipids influence their mobilization from the aqueous to particulate (membrane) compartments. Thus, there are proteins that bind to membranes through post-translational lipid modifications (or covalent-lipid proteins, CLPs) and others that bind through hydrophobic amino acid domains (or lipid-binding proteins, LBPs). Interestingly, both types of interactions can be regulated, given that some covalent modifications that influence the way proteins are sorted to different organelles or membrane microdomains are reversible (e.g., S-palmitoylation). This fatty acylation regulates transmembrane protein transport through the Golgi apparatus [31], protein–lipid raft interactions [32], the activity of G-protein coupled receptors (GPCRs), and other membrane receptors [33]. Membrane lipid structure can also modify these interactions as addressed below.

Palmitoylation also affects peripheral proteins, such as G proteins [34], and interestingly, most G protein alpha subunits (Gα) are reversibly modified by palmitoylation [35]. Moreover, G protein activation by GPCRs has been associated with increased palmitoylation, and the turnover and activation of the Gαs subunit produces its reversible mobilization from the plasma membrane to the cytosol [36].

**Figure 1:**
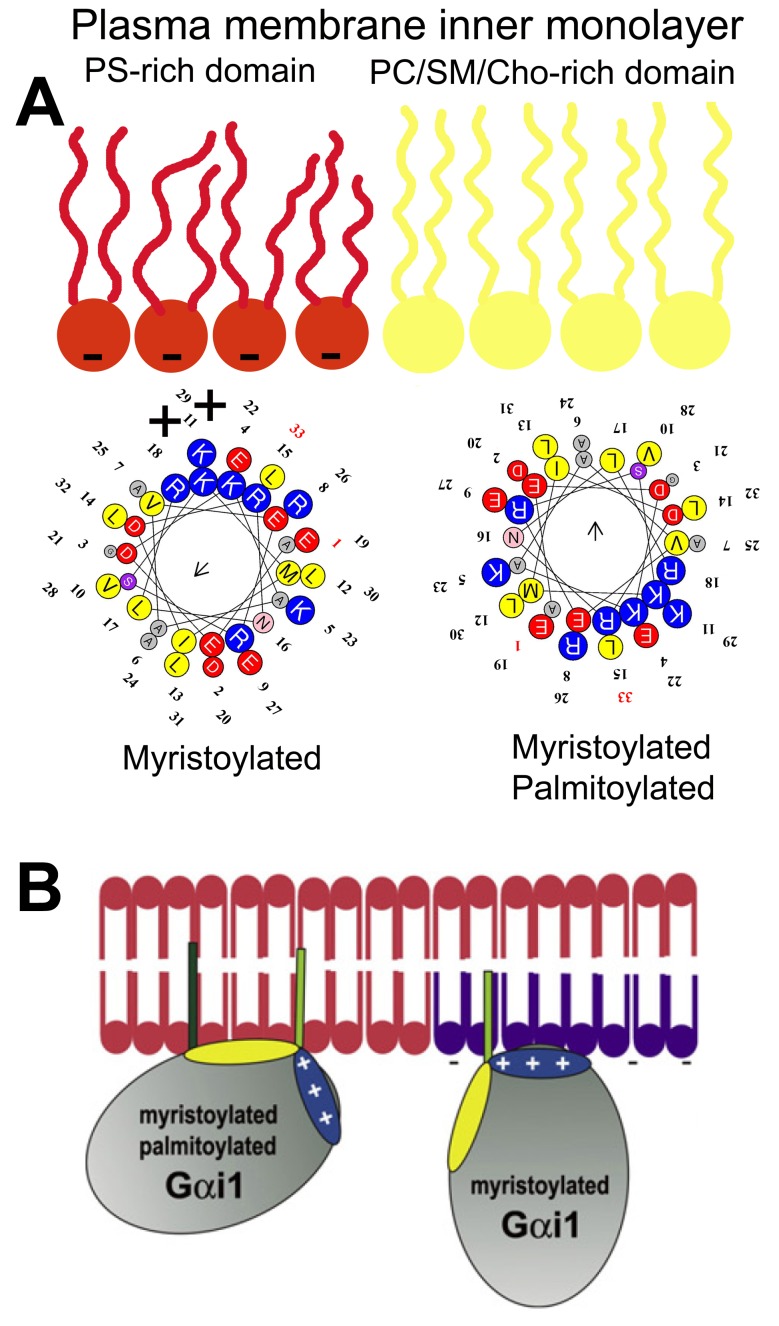
Gαi_1_ protein–membrane interactions. (**A**) Geometry of the N-terminal α-helix with a myristoyl moiety interacting with PS-rich (negatively charged, red) membrane microdomains (left helix), and the α-helix with myristoyl and palmitoyl moieties (right helix) that interact with PC and/or SM and/or Chol microdomains (yellow). (**B**) Scheme of acylated Gαi_1_ protein–membrane interactions (Adapted from [9]).

Inactive Gα subunits localize to non-lamellar-prone membrane microdomains that are rich in PE, where they bind to Gβγ dimers to form Gαβγ heterotrimers [23]. In contrast to lipid rafts, these PE-rich microdomains form liquid disordered (Ld) bilayer regions due to the low surface lipid packing and high acyl chain mobility [8,37]. The strong affinity of the Gβγ dimer for these microdomains facilitates the piggyback transport of the Gα subunit towards GPCRs, facilitating their productive interaction with activated receptors [23]. The G protein activation induces a dissociation of the Gα subunit from the Gβγ dimer. These Gα monomers have a preference for lipid raft (liquid ordered, L_o_) microdomains, and they are mobilized to raft-like microdomains where they interact with signaling effectors, including adenylyl cyclase, guanylyl cyclase, phospholipase C, ion channels, etc. [9,23,24]. Both electrostatic and hydrophobic interactions participate in this peripheral protein mobilization from one membrane microdomain to another. In this scenario, Gαi_1_ protein palmitoylation is critical for its translocation from phosphatidylserine (PS)-rich microdomains to microdomains with a high lamellar propensity, mainly rich in phosphatidylcholine (PC) and/or sphingomyelin (SM) and cholesterol (Chol: [9]). This is due to the fact that the N-terminal α-helix of the Gαi1 protein interacts with negatively charged (PS-rich) membrane microdomains through a positively charged amino acid patch when the protein is myristoylated but not palmitoylated [9]. However, the palmitoylated Gαi_1_ protein N-terminal region interacts through an aliphatic or positively charged amino acid patch which induces monomeric G protein mobilization from negatively charged membrane microdomains to neutral phospholipid regions (Figure 1).

Thus, fatty acyl moieties in peripheral (amphitropic) membrane proteins not only serve as anchors for their binding to membranes but also, they drive their mobilization from one membrane microdomain to another. The relevance of membrane microdomains is that they form “clubs” with a lipid composition that attracts different types of proteins. Proteins form nanoclusters in different membrane lipid domains that exert physical interactions with signaling partners, resulting in productive signaling under the correct circumstances. For signal amplification, a huge number of G proteins must co-exist with GPCRs, a phenomenon mediated by protein–lipid interactions. Although protein–protein interactions for GPCR-G protein coupling have been studied intensely (e.g., [38]), the crucial role of lipids in this binding is not fully understood. In this context, GPCRs have G protein-subtype binding preferences, although this coupling is not truly specific. One type of GPCR can bind to different types of G proteins with similar or different affinities, and one G protein subtype can be activated by different GPCRs [39]. Therefore, differences in expression in defined cells or variations in lipid composition, which could regulate receptor-G protein interactions, have important consequences for cell signaling. This is especially important during the pathophysiological and/or therapeutic regulation of the plasma membrane lipid composition, as lipid modifications alter protein–membrane interactions, the propagation of cell signals and the cell’s physiology (and even the regulation of gene expression [20,21,40]).

**Figure 2:**
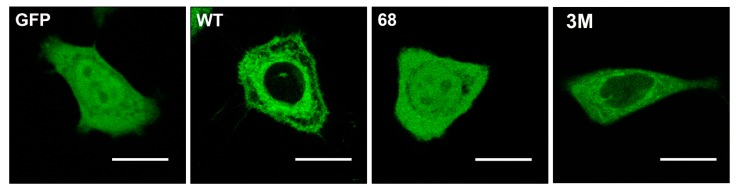
Gγ2 protein–membrane interactions. The green fluorescent protein (GFP), containing the wild-type C-terminal region of the Gγ2 protein (WT), shows a membrane localization that does not coincide with that of GFP alone (GFP). Mutations that alter the presence of the isoprenyl moiety (68: C68S) or the 3 C-terminal basic amino acids (3M: R62G, K64G, K65G) have a huge impact on the distribution of the protein in SF-767 cells. Bar = 15 μm (adapted from Reference [24]).

Other protein anchors frequently found in peripheral CLPs are isoprenyl moieties. This type of modification involves adding farnesyl or geranylgeranyl residues to the C-terminal regions of signaling proteins like Ras, Gγ protein, Cdc42, Rho, Rac, etc. Farnesyl (FTase) or geranylgeranyl transferase (GGTase) catalyze the prenylation of the cysteine residue of the C-terminal CaaX box, where “a” refers to an aliphatic amino acid and “X” to any amino acid [41]. When “X” is Leu, then the protein is gerenylgeranylated, whereas the protein is farnesylated if “X” is Ser, Ala, Cys, Gly, Thr, His, Asn or Gln, and “X” can be modified by both enzymes when it is Met, Val, Ile or Phe [41,42,43,44]. In addition, the CaaX box is also subject to proteolysis by the Ras converting enzyme 1 (RCE1) which removes the last three amino acids (aaX: [45,46], and to methylation by isoprenyl carboxyl methyltransferase (ICMT) that further increases hydrophobicity of the C-terminal region [47,48]. The processing of peripheral signaling proteins with a CaaX motif influences their localization. Thus, C68 mutations in Gγ2 protein:GFAP chimeras alter the localization of this fusion protein from the membrane to a more homogeneous cell distribution (Figure 2) [24]. Interestingly, mutation of the polybasic cassette (R62, K64, and K65) also causes mislocalization of the Gγ_2_ protein but with a different pattern to that of the Cys mutations (Figure 2) [24].

In general, the formation of membrane microdomains with specific lipids favors the presence of certain peripheral proteins, while hindering the interaction of other proteins. For example, caveolae (“little caves”) form spatio-temporal platforms where EGFR, Ras, and Raf1 meet to propagate signals promoting cell growth [49]. Similarly, L_o_ microdomains (e.g., lipid rafts) are preferred by Gαi_1_ proteins, whereas L_d_ microdomains bind with high affinity to Gαβ and Gαβγ proteins [9,23]. Moreover, the co-operative binding of Gαi_1_ proteins to lamellar-prone L_o_ membranes is in part due to the presence of myristoyl or palmitoyl moieties [29]. The presence of fatty acyl moieties in GPCR nanoclusters produced by the presence of G proteins regulates membrane lipid structure in a way that also enhances the binding of Gαβ and Gαβγ proteins to non-lamellar-prone L_d_ microdomains but not to L_o_ microdomains. By contrast, the presence of farnesyl or geranylgeranyl moieties in lipid bilayers favors the co-operative binding of Gαβ and Gαβγ proteins to Ld membranes while inducing dramatic reductions in Gαi1 protein binding to membranes [29,50].

**Figure 3:**
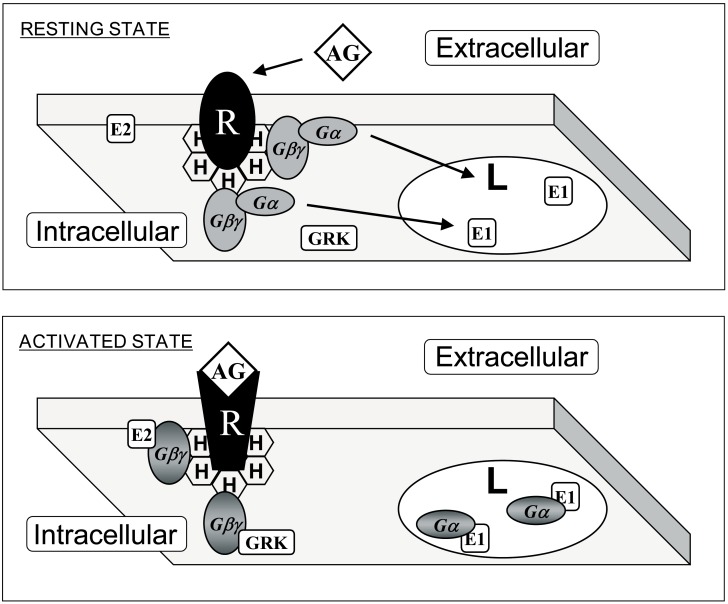
Interaction of G proteins with membrane microdomains. **Upper panel**: G protein-coupled receptor (GPCR, R) and heterotrimeric G (Gαβγ) proteins prefer membrane microdomains rich in non-lamellar-prone (H) lipids. This effect is driven by prenylated Gαβ dimers. **Lower panel**: Upon receptor-induced activation, acylated Gα subunits are mobilized to lamellar-prone membrane microdomains (L). In addition, localization to phosphatidylserine-rich or -poor domains is controled by a polybasic domain exposed to the membrane, and not by the presence or absence of a palmitoyl moiety (see Figure 1). AG: agonist; E1 and E2: effector protein 1 and 2: GRK: GPCR Receptor Kinase. Adapted from Reference [23].

In addition to isoprenyl or acyl moieties that are found frequently at the C- or N-terminal domains of amphitropic signaling proteins, a polybasic domain is also found flanking these lipidated amino acids [9,24,51]. These positively charged amino acid clusters, mainly containing Arg or Lys residues, define the preference of these proteins for lipid microdomains rich in PS or other negatively charged lipids, with relaxed specificities, as well as participating in the mobilization of proteins between membrane microdomains (Figure 1 and Figure 3). In this context, the dynamics of proteins containing polybasic domains and of membranes with negatively charged amino acids depends largely on electrostatic interactions. Therefore, membrane areas with a high PS content attract polybasic amino acid-containing proteins, yet they do not restrict their movement as tightly as membranes with PtdIns(4,5)P2 [52]. This phenomenon suggests that proteins which prefer membrane microdomains rich in monovalently charged PS may be more able to move among membrane microdomains than those proteins that interact with polyvalent anionic lipids like PIP2.

Other amino acids also participate in CLP–membrane interactions. For example, hydrophobic amino acids and motifs are involved in the reversible or permanent interaction of proteins with membranes. One such interaction occurs with the transmembrane domains of integral membrane proteins. In this context, many structures provide permanent protein anchorage to membranes. Proteins with only one transmembrane domain have been found, such as Notch [53], or with multiple domains like the proton-coupled folate transporter [54]. These transmembrane regions frequently form α-helices, as the GPCRs [55], although some β-barrel structures have also been found like those found in porins [56].

However, this work focuses on the interactions of non-permanently bound membrane proteins, a scenario in which a number of amino acid sequences are known to be involved in reversible interactions of amphitropic signaling LBPs with membranes. For example, the C1 and C2 protein domains have been thoroughly studied, the former members of the Cys-rich protein superfamily that are classified as typical if they bind to DAG and phorbol esters or atypical if they do not [57,58,59]. This amino acid domain is especially interesting in relation to protein kinase C (PKC), as it may be a dual C1A/C1B domain in classic and novel PKC isozymes (α, β, α, δ, θ, ε, and η) or a single domain (C1) in the atypical PKCs (ι/λ, ζ: [58,59,60]). Interestingly, DAG induces negative curvature strain in membranes and it favors the appearance of non-lamellar (H_II_) lipid structures in vitro [61], a biophysical property of membranes that regulates PKC binding [8,14]. In biological membranes, DAG reduces the surface packing strain and the polar head lateral pressure, which allows specific protein and lipid insertion. When non-lamellar phase propensity is altered by pathophysiological processes or therapeutic agents, the interaction of PKC (and of other peripheral proteins) is affected, inducing relevant changes in its interaction with the plasma membrane [8,14]. The C1 domain also recognizes tumor promotor phorbol esters, which induce non-lamellar phases but in a different manner to that caused by DAG or PE, such that PKC was associated with conditions related to cell growth, as in cancer [62,63], AD [64], CVDs [65], and immunological diseases [66], etc. Thus, not only do PKC–membrane interactions define relevant signaling events, but they also may be involved in pathophysiological and therapeutic processes [8,14]. Initially identified in PKC, the DAG and phorbol ester responsive C1 domain was subsequently described in other protein families: the Unc-13 scaffolding proteins, MRCKs, RasGRP proteins, protein kinase D, chimaerins, and the β and γ DAG kinase isoforms [60]. Protein–membrane interactions are crucial to therapies and the potential use of the C1 domain as a drug target has already been proposed [67]. Like the C1 motif, the C2 motif is found in numerous eukaryotic proteins involved in cell signaling and its conserved sequence serves as a membrane docking motif [68]. It has been found in many relevant proteins, including PKCα [69], synaptotagmin [70], phospholipase Cα [71], and cytosolic phospholipase A2 [72]. The C2 domain is involved in the binding of PKCα to PS, an important phospholipid in the inner leaflet of the plasma membrane [69]. In classic but not novel PKC isozymes, Ca^2+^ ions are involved in this binding, which may also occur with other negatively charged phospholipids [73]. In addition, this domain can also bind PIP2 due to the fact of its lysine residues [59,73]. The phosphoinositide binding site is located in the β3-β4 strands of the C2 domain in PKCα [74]. Over one hundred C2 domains have been reported in proteins with a wide range of signaling functions, covering protein phosphorylation, vesicular transport, lipid modifications, GTPase regulation, etc. [74]. Despite the homology among these C2 domains, particular structural differences drive their diverse protein–lipid interactions, suggesting that specific therapies could be targeted to C2 domains.

Other motifs and domains have been described that favor the interaction of LBPs with membranes. For example, myelin basic protein (MBP) exerts electrostatic interactions between positively charged amino acids and negatively charged phospholipids at the plasma membrane [75]. Many peripheral proteins containing poly-lysine clusters, and like the aforementioned G proteins, they display electrostatic interactions with negatively charged phospholipids that are modulated by other membrane lipids [76]. In the case of MBP, it binds around Chol-rich membrane microdomains in which the presence of SM is required for C1 variants of the MBP but not for C8 variants. This fact appears to be related to multiple sclerosis (MS) in which more MBPs are found in the brains of patients where SM levels are lower relative to healthy adults.

In addition to these lipid-binding domains, other protein motifs mediate interactions with membrane lipids. For example, the spectrin homology 3 (SH3) domain is common to signaling proteins that mediate protein–protein interactions through binding to proline-rich sequences. However, SH3 domains may also be involved in protein–lipid interactions, such as in caskin1, which is involved in binding to lysophosphatidic acid (LPA) and sphingosine-1-phosphate lipids [77]. Similarly, the pleckstrin homology (PH) domain of phosphoinositide-dependent kinase 1 (PDK1) is involved in protein–lipid interactions and it was suggested that it might constitute an alternative drug target rather than the catalytic site [78]. In addition, sterile alpha motifs (SAMs) are amino acid regions usually involved in protein–protein interactions. Nevertheless, they have also been seen to participate in protein–membrane interactions in the p73α protein due to the fact of its capacity to bind to membrane phospholipids [79]. All these protein regions are involved in the interactions with biological membranes that regulate cell signaling and that represent potential druggable motifs [80]. This approach is one of the potential means to develop therapies based on the control of the membrane lipid bilayer and the signals they regulate (i.e., MLT [21]).

## 3. How Membrane Lipid Structure Influences Protein–Lipid Interactions

In general, proteins can interact with membranes via interactions with specific lipid species, with membrane lipid structures, electrostatic, hydrophobic interactions, etc. Peripheral membrane proteins can use more than one of these strategies to bind to membranes, which favors their versatile localization to membrane microdomains or cell compartments, which depends on the membrane lipid composition. Numerous studies have defined the interactions between peripheral proteins and specific membrane lipids, some of which were described above. A wide range of proteins have been shown to interact with Chol, phosphoinositides, PS, SM, free fatty acids (FFAs), etc. However, this section aims to review the interaction of amphitropic signaling proteins with membrane structures rather than with specific lipid species. This type of interaction deserves further attention because: (i) the plasma membrane is a critical hub for signaling proteins; (ii) cells can regulate their lipid composition according to a range of pathophysiological situations; (iii) membrane lipids organize into different microdomains rich in specific lipid species, which attract different types of proteins; and (iv) proteins that prefer certain types of lipid structures can drive productive interactions involving the reception and propagation of cell signals [8,9,20,23,37]. The interaction of the non-permanently bound membrane proteins, G proteins and PKC, with non-lamellar-prone (H_II_) membrane structures was first described some years ago [8]. In this context, one of the mechanisms of action by which anthracyclines exert their antitumor action was through the inhibition of H_II_-phase propensity and the subsequent mislocalization of these signaling proteins. This phenomenon explained why anthracyclines could kill cancer cells solely by interacting with the plasma membrane but not entering the cells [81]. Subsequently, important modifications of the plasma membrane’s lipid composition by anthracyclines was seen to be relevant to their mechanism of action [12].

The plasma membrane is a critical element in cell signaling, as almost all incoming or outgoing messages must cross this barrier. Changes in the plasma membrane lipid composition and structure are involved in numerous physiological and pathological phenomena. For example, the body temperature of fish that live in rivers undergoes important variations since the water temperatures can range from 4 to 20 °C. In these fish, important temperature-dependent changes in phospholipid species and fatty acyl chains can be seen [82,83]. Membrane lipid structure is temperature dependent, changing in accordance with the temperature of the water in fishes [84]. The changes in lipid species in the brain of cold-water fishes between summer and winter maintain membrane fluidity, and other biophysical properties of membranes, constant, which in turn ensures correct protein function and cell signaling in these animals [82,83]. If temperature and lipid composition can alter membrane lipid structure and cell signaling, pathophysiological changes and pharmaceutical/nutraceutical interventions that regulate membrane lipid composition may also influence the health of cells.

Heterotrimeric G proteins were thought to prefer L_d_ membrane microdomains when extracted from the rat brain [37]. Using purified G protein monomers (Gα), dimers (Gβγ), and trimers (Gαβγ), the Gαβ heterodimer was seen to drive the interaction of Gαβγ heterotrimers with membranes [23]. Thus, one of the roles of Gαβ dimers is to bring Gα monomers into contact with GPCRs. Using G protein mutants, we described the molecular basis of these interactions for different G protein subunits [9,24]. Membrane microdomains rich in PE form liquid disordered (L_d_) membrane microdomains, which differ in their lipid composition and structure from lipid rafts, caveolae, synaptosomes, and other types or membrane lipid microdomains. The localization and activity of important peripheral signaling proteins is very sensitive to changes in membrane structure [8]. Therefore, natural or synthetic molecules that regulate lipid polymorphism in vitro and membrane microdomains in vivo [85] can regulate the localization and activity of peripheral membrane proteins, and thereby modulate cell signaling.

Cell membranes favor the formation of multiprotein complexes, in which certain proteins receive signals, other proteins act as scaffolds, and others participate in signal propagation or second messenger production. The protein complexes involved in signal propagation are frequently called signalosomes, and their arrangement and activity is very dependent on membrane lipid composition, altering the protein–lipid interactions for example those involved in AD [86]. Moreover, sphingolipids appear to be critical in the prognosis of anaplastic lymphoma. Thus, ALK+ lymphomas may express an ALK fusion protein involved in cancer cell survival, or the Cbp/PAG adaptor protein and the Lyn kinase signalosome that recruits other transcription factors and signaling enzymes. Lyn is not particularly active in ALK+ lymphoma membranes that contain sphingolipid-rich domains (i.e.: raft-like membrane microdomains) which impairs the productive signaling of the Lyn-Cbp/PAG signalosome [87]. Similarly, the lipid composition of membranes is critical for the binding of isoprenyl-bearing proteins to membranes and for the subsequent propagation of messages from receptors to effectors as well as for the propagation to further signaling proteins like transcription factors [14,23,24,40,88]. Therefore, the plasma membrane appears to act as a switch, and alterations in its composition cause dramatic translocations of proteins to or from the plasma membrane (see below). Such signals appear to be especially relevant in the context of cell proliferation. Thus, either the increase in cell proliferation caused by tumor alterations or decreased proliferation related to neurodegeneration (e.g., AD or Parkinson’s disease (PD)) have been related to membrane lipid modifications [8,12,15,88,89].

As described above, membrane microdomains act as sites where signaling partners exert productive interactions. As such, signaling proteins can interact with downstream signal transducers, sharing their affinity for certain membrane lipids or lipid structures. Lamellar-prone L_o_ membrane microdomains (e.g., lipid rafts or caveolae) contain specific lipids that define their membrane lipid structure and that are involved in selecting the proteins that bind to them [9,90]. The ability of lipids to organize into different structures (lipid mesomorphism) depends on the lipid composition and external physical factors, such as temperature. The mosaic of lipid structures that defines different membrane microdomains facilitates a number of different protein–lipid interactions [20,21,91]. Moreover, peripheral proteins modulate the membrane lipid organization, and, thus, they can regulate the interaction of amphitropic signaling proteins with membranes [29]. A very interesting case is the effect of myristic acid, palmitic acid, and isoprenyl moieties covalently bound to proteins. These post-translational modifications regulate the membrane structure in a way that facilitates the co-operative binding of protein molecules that bear these modifications. Thus, myristoyl and palmitoyl moieties favor the binding of G proteins to lamellar-prone (L_o_) membrane microdomains, and isoprenyl moieties favor their binding to non-lamellar-prone (L_d_) membrane microdomains [29]. In addition, myristoyl and palmitoyl moieties favor L_o_ lipid structures while isoprenyl moieties favor L_d_ lipid structures [29]. Non-lamellar-prone lipids also regulate the membrane shape, and negative Gaussian curvature enhances the activity of DGKe and controls the PtdIns-cycle at endoplasmic reticulum-plasma membrane contact sites [92,93]. Given the relevance of PtdIns in cellular functions, the membrane shape appears to be a critical parameter in the cell’s physiology. In fact, membrane structures with special shapes, such as membrane contact sites, synaptosomes, caveolae, etc., host important function that require bilayers with differential biophysical properties.

Another well-studied protein–lipid interaction is the Ca^2+^-mediated fusion of synaptic vesicles to membranes in order to release neurotransmitters into the synaptic cleft. In this process, Ca^2+^ binding to the C2 domain of synaptotagmin mediates vesicle exocytosis, assisting fusion to the plasma membrane via its interaction with a SNARE/complexin complex in presynaptic terminals [94]. In general, non-lamellar-prone membrane microdomains rich in PE or DAG are necessary for interactions with the C2 domain in certain proteins. Moreover, they are necessary for membrane fusion and fission phenomena, such as exocytosis and endocytosis, which require the formation of inverted curvature non-lamellar (H_II_) intermediates [95,96,97].

An important feature of biological membranes is transbilayer lipid asymmetry, which influences both membrane physical properties and protein lipid interactions [98]. Thus, higher levels of SM and PC have been found in the outer plasma membrane leaflet, whereas PE and PS are more abundant in the inner leaflet [99]. This asymmetry has a relevant impact on protein–membrane lipid interactions and, indeed, the number of peripheral proteins bound to the inner leaflet is higher than that bound to the outer leaflet [100]. Transmembrane asymmetry is also observed in other cell membranes, such as the mitochondrial outer membrane, which causes differential biophysical properties that affect permeabilization and protein–membrane interactions [101].

In summary, membrane lipid composition plays a crucial role in the interaction of peripheral membrane proteins with the lipid bilayer which is mediated by the binding of these signaling proteins to specific lipid species and to supramolecular membrane structures, known as membrane microdomains. Microdomains like synaptosomes, caveolae, lipid rafts, liquid disordered domains, etc., act as signal propagation platforms where signaling partners have a higher probability of physically interacting.

## 4. Altered Membrane Lipid and Amphitropic Protein Interactions in Human Diseases

**Figure 4:**
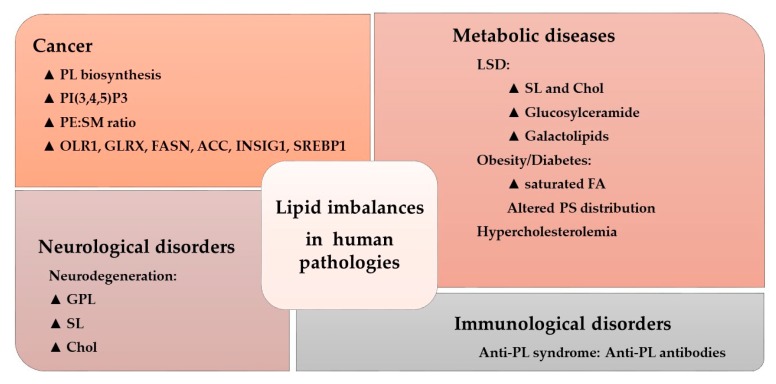
Lipid imbalances and human pathologies. Alterations to the lipidome in a variety of conditions. The triangle indicates increased levels or pathway activity: PL, phospholipid; PtdIns(3,4,5)P3, phosphatidylinositol 3,4,5-trisphosphate; PE, phosphatidylethanolamine; SM, sphingomyelin; OLR1, oxidized low-density lipoprotein receptor 1; GLRX, glutaredoxin; FASN, FA synthase; ACC, acetyl-CoA carboxylase; *INSIG1*, insulin induced gene 1; SREBP1, sterol regulatory element-binding protein 1; LSD, lysosomal disorder; SL, sphingolipid; Chol, cholesterol; FA, fatty acid; PS, phosphatidylserine (Adapted from [30]).

The activity of many amphitropic proteins depends on their membrane interactions, which are modulated by the lipid composition of the membrane. The activity of several important signaling proteins is regulated by protein–lipid interactions, including Src kinase, RAS-guanine nucleotide exchange factor, cytidylyltransferase, PKC, phospholipase C, vinculin, and DnaA protein. Therefore, alterations to membrane lipids can have an important influence on several diseases. For example, cystic fibrosis causes lipid imbalances that affect surfactant function, producing a negative effect on breathing [102,103]. In mouse models of cystic fibrosis, a similar lipid imbalance was found in affected organs, although administration of docosahexaenoic acid (DHA) normalized both these lipid changes and the animal’s health status [104]. In brain injury, a significant increase in SM, PE, PC, and the derivatives lysoPE and lysoPC have been described at acute and/or sub-acute time points [105]. Likewise, distinct lipid imbalances have been associated with other pathologies, as recently reviewed (Figure 4) [30].

These alterations could modify the activation of amphitropic proteins. In diabetes, DAG levels are chronically elevated in various tissues, such as the retina, aorta, heart and renal glomeruli, liver and skeletal muscles, leading to abnormal PKC activation [106]. The PKC exists in a cytosolic auto-inhibited latent form or in a membrane-associated active form. Its membrane recruitment is accompanied by a conformational rearrangement that relieves auto-inhibitory interactions, enabling PKC to bind to membranes through its C1 and/or C2 domains, and allowing it to phosphorylate its targets [107,108]. A large number of human diseases are related to alterations that affect PKC isoenzymes. For example, PKCα is a major regulator of heart contractility [109], platelet aggregation in thrombosis [110], and it has been implicated in virtually every stage of the development of atherosclerotic disease [111]. In ischemia and reperfusion injury, PKCδ and PKCε have been attributed opposing roles [112], and PKCα is altered in hypertensives subjects [25]. Moreover, altered phospholipid metabolism has been well documented in several neurological and psychiatric diseases [113], including AD, in which PKC signaling is critical for the non-toxic degradation of the amyloid precursor protein (APP) and the inhibition of GSK3β, which controls tau phosphorylation. In addition, misregulation of PKC signaling may be involved in the origin of AD [114]. Moreover, alterations to PKC have also been described in the brain of heroin addicts [115]. PKC activity also plays an important role in cancer, having been described as both a tumor promoter and tumor suppressor, as reviewed in depth elsewhere [116]. Interestingly, PKC-membrane interactions are also involved in the mechanism of action of certain antitumor drugs [8,14]. The next sections review protein–lipid interactions in important pathologies and therapeutic approaches based on the regulation of such interactions.

## 5. Protein–Lipid Interactions in Cancer

The RAS family of amphitropic proteins are mutated (especially K-RAS) in 95% of pancreatic, 45% of colorectal, and 35% of lung cancers [117]. Guanine nucleotide exchange factors (GEFs) and GTPase activating proteins (GAPs) control RAS activation by inducing GDP exchange for GTP or GTP hydrolysis to GDP, respectively. To regulate RAS activation, GEFs and GAPs are recruited to plasma membrane microdomains close to RAS. The activity of K-RAS has been directly related to membrane regions rich in PS, which interact with a polybasic amino acid region in the C-terminal region of this protein [118].

The lipids, SM and Chol, participate in the formation of membrane rafts that attract specific proteins which regulate relevant cellular events, such as cell death or cancer cell differentiation [119]. In this context, palmitoylation mediates the affinity of a protein for lipid rafts. For example, S-palmitoylation of the Gαi_1_ protein regulates its interaction with lipid rafts and affects its membrane localization [9]. In the case of H-RAS and K-RAS, farnesylation, and subsequent palmitoylation regulate their trafficking between the Golgi complex and the plasma membrane [120], and their ensuing signaling efficiency [121]. Palmitoylation of RAS occurs at Golgi membranes and it drives RAS to the plasma membrane via vesicle trafficking [122]. The presence of RAS at the plasma membrane is necessary for its activity as a tumor promoter, which depends on its covalent acylation. But palmitoylation is not only important for RAS activity, it is also essential for the function of other oncogenes (e.g., *EGFR*) and tumor suppressors (e.g., SCRIB, melanocortin 1 receptor).

The Wnt signaling pathway regulates a variety of cellular processes, including differentiation, proliferation and stem cell pluripotency. Hence, aberrant activation of the Wnt-FZD signaling leads to tumorigenesis in many tissues [123], including the breast [124], prostate [125], colon, brain [126], and pancreas [127,128]. In this context, Wnt proteins are modified by lipidation, a post-translational addition essential for their activity in both normal and cancer cells [129]. Members of the Wnt family undergo two types of post-translational modification that influence their interactions with lipid bilayers and that are essential for Wnt signaling: serine acylation and the subsequent S-palmitoylation of cysteine. Wnt signaling involves crosstalk with other important cell signaling pathways including the Notch, Hedgehog, and EGFR cascades [130], all of them altered to some degree in different cancers [131,132,133]. All these key signaling regulators are controlled by lipid–protein interactions, which highlights the relevance of these interactions in cancer. Accordingly, modulation of these lipid–protein interactions may produce potential therapeutic benefits in the treatment of cancer [8,20,134,135]. This approach has been termed MLT or melitherapy, and it has been demonstrated to combine high efficacy and safety in clinical trials (e.g., ClinicalTrials.gov identifiers NCT01792310 and NCT03366480).

## 6. Protein–Lipid Interactions in Neuroregeneration

Neurodegenerative diseases are a public-health issue worldwide and these are conditions with relevant unmet clinical needs. Classic therapies focus on preventing or delaying neuronal degeneration, whereas more recent interest has also focused on neuroregenerative therapies. For a long time, there has been a strong movement towards understanding the requirements of Neural Stem/Progenitor Cells (NSPCs) for therapeutic goals. The finding that NSPCs persist in adults, and the discovery of relevant transcription factors and signaling pathways, including signaling lipids that influence NSPC behavior and of neurogenesis, raised hope in therapies based on NSPC regulation and the potentiation of neurogenesis [136]. In this section, we will show that lipid alterations and protein–lipid interactions are involved in the development of neurodegenerative diseases and their therapy [15,137].

Lipid metabolism regulates the proliferation of NSPCs. Cholesterol is one of the most abundant lipids in the central nervous system (CNS), and as Chol deficits can alter brain development, its effects during this period have been studied widely [138]. However, how Chol affects NSPCs is poorly understood. Interestingly, decreased Chol biosynthesis has little effect on NSPC proliferation when compared to its effect on newborn neurons, which undergo massive death by apoptosis when Chol is limited. Similarly, the radial glial network that supports the migration of newborn neurons also seems to be affected by changes in Chol [139,140]. Consequently, reduced Chol levels at cell membranes seems to compromise brain neurogenesis and NSPC migration. Weaker Chol biosynthesis affects the mitotic behavior of NSPCs and it induces premature differentiation into neurons, which could explain why newborn neurons undergo apoptosis in these conditions [140]. Interestingly, these defects can at least be partially prevented by feeding pregnant animals Chol supplemented diets [140]. In addition, Chol biosynthesis compromised NSPCs contain more intracellular lipid droplets and increased VEGF (Vascular Endothelial Growth Factor), the latter promoting angiogenesis [139]. Both events suggest that impaired Chol biosynthesis in NSPCs activates compensatory mechanisms for Chol-lipoprotein uptake from the blood. Therefore, the widely described defects in brain development could be a consequence of the NSPC dysregulation induced by Chol depletion in NSPCs, newborn neurons and the glial fiber network. This evidence indicates that cell membrane Chol regulates neurogenesis and neuronal cell migration.

Polyunsaturated fatty acids (PUFAs), like DHA (C22:6, n-3) and AA (arachidonic acid, C20:4, n-6), are abundant in the CNS. Their conversion from essential precursors is very poor in humans and they are mostly obtained from dietary sources. Lipids are indeed abundant in the CNS and the brain is the organ with the highest DHA levels [141]. Botth DHA and AA are involved in many signaling cascades, both acting as precursors for docosanoids and eicosanoids that fulfil different roles in the cell [142,143]. Studies reviewing the effect of these PUFAs on NSPC regulation support a role for both of them in neurogenesis during brain development and adulthood. Specifically, AA increases NSPC proliferation, and it probably influences the maintenance of the NSPC pool, whereas DHA promotes neuronal differentiation [144,145]. However, not only do the individual levels of these two PUFAs in cell membranes play a role in neurogenesis but also, the ratio between them is determinant as a lipid switch. Interestingly, the experimental combination of both PUFAs did not outperform individual enrichment which suggests that an optimal ratio of omega-3 and omega-6 PUFAs (i.e., DHA:AA) must be established to enhance neurogenesis [146]. Therefore, modulation of these PUFA levels and the omega-3 to omega-6 ratio may constitute an adjustable lipid switch that can turn-off pathological neurodegeneration [147]. Due to the higher proportion of omega-6 PUFAs in western diets, low dietary omega-6/omega-3 ratios have been widely described as beneficial on diverse pathologies.

In general, PUFAs have unique biophysical properties in membranes, regulating their interactions with proteins. They favor the occurrence of L_d_ membrane microdomains [85,148], which are associated with changes in protein–lipid interactions. In this context, a decline in DHA biosynthesis correlates with cognitive impairment in AD patients [149]. Alterations to membrane lipids in neurons have been proposed as upstream events that later generate molecular changes implicated in neurodegeneration, such as Aβ production and tau phosphorylation. These lipid alterations might affect protein–lipid interactions that would activate the neurodegenerative cascade, as well as modulating neuroprotection and neuroregeneration [15]. Indeed, treatment with the PUFA 2-hydroxydocosahexaenoic acid inhibits amyloid production, tau phosphorylation, and it induces an increase in PUFAs and the recovery of cognitive scores in a mouse model of human AD (5XFAD mice: [15]).

Lipids regulate NSPC proliferation, migration and differentiation through a variety of mechanisms, one of which involves lipid raft modulation. These membrane microdomains are implicated in the cell signaling pathways that regulate stem cell maintenance, such as the EGF (endothelial growth factor), insulin, and Wnt/β-catenin pathways [150,151]. In this context, both Chol and PUFAs can modulate lipid-raft-mediated signaling by regulating the composition of these structures [21,147]. For instance, increased levels of cell membrane PUFAs are associated with increased NSPC proliferation due to the disruption of protein localization to lipid rafts [152]. Membrane lipids can also regulate signaling in NSPCs through FA binding to specific receptors, such as FABPs (fatty acid binding proteins). Three members of this family are expressed in the brain: FABP3, FABP5 and FABP7 [153]. The protein FABP3, is related with neuritogenesis and synaptogenesis, whereas FABPs 5 and 7 are involved in NSPC differentiation and migration [154]. Interestingly, FABP7 may regulate the Pax6 and Notch signaling cascades, which are highly relevant in NSPCs [155,156]. Other receptors influenced by DHA and other PUFAs and that are involved in neurogenesis have also been described. Thus, DHA has been shown to bind (directly or via FABPs) to PPARγ (peroxisome proliferator-activated receptor γ), a nuclear receptor that mediates the expression of transcription factors that enhance neurogenesis [157]. The PUFA omega-3, DHA, also binds to GPR40 (G-protein coupled receptor 40), the activation of which generates DAG and IP3, a signaling molecule produced from the lipid PtdIns that mediates Ca^2+^ release from the ER, leading to the neuronal differentiation of NSPCs [158]. Although many activities mediated by PUFAs can be attributed to their influence on membrane structure and the ensuing regulation of protein–lipid interactions, PUFAs like DHA and AA may also exert their physiological roles via bioactive metabolites. These metabolites may also bind to cellular receptors to modulate neurogenesis. For instance, the DHA endocannabinoid-like metabolite synaptamide and the hydroxylated DHA-derivative NPD1 (neuroprotetin D1) both promote the neuronal differentiation of NSPCs [143].

## 7. Lipid–Protein Interactions in Diabetes

Insulin resistance has been widely associated with an altered cell membrane composition, particularly in Type-2 diabetes mellitus (T2DM). Insulin resistance is characterized by a restriction in the ability of insulin to exert its physiological functions in tissues, leading to insulin hypersecretion by the pancreas as a compensatory mechanism to maintain glucose homeostasis. Unfortunately, this hyperinsulinemia induced by insulin resistance contributes to pancreatic β-cell failure and the further development of diabetes [159].

Cell lipids are essential regulators of insulin sensitivity since changes in the dynamic properties of the cell membranes (e.g., membrane fluidity) in part lead to insulin resistance. For example, membrane viscosity is enhanced, and insulin resistance increased in the liver of insulin-resistant animals. A similar relationship was also found in humans, where an increase in PUFAs augments membrane fluidity, which has been associated with upregulated insulin sensitivity in skeletal muscle membranes [160]. Cell membranes enriched in saturated fatty acids and Chol (common dietary components in industrialized countries) are prone to form L_o_ membrane microdomains, such as lipid rafts, which promote membrane rigidity and viscosity. By contrast, enrichment in monounsaturated fatty acids (MUFAs) and PUFAs promotes the formation of L_d_ membrane microdomains, favoring membrane fluidity [148]. In this context, one variant of the raft domains are caveolae structures. Caveolin is an insulin receptor (IR) activator, the latter requiring the scaffolding activity of the former to become activated in caveolae microdomains [161].

IR activation and its affinity for insulin depends on the cell membrane composition and structure. Decreased membrane fluidity caused by a high saturated FA content leads to less IR in the plasma membrane and reduced insulin affinity. However, the presence of PUFAs (particularly omega-3 PUFAs like DHA) increases membrane fluidity and insulin sensitivity [162]. Moreover, increases in GM3 (a ganglioside strongly associated with lipid rafts) promote IR depletion from lipid rafts, disrupting insulin signaling [163,164]. Accordingly, insulin signaling is enhanced in mice lacking GM3 synthase [165]. In addition, IR can form complexes independently with Caveolin 1 and GM3, and insulin signaling depends on IR localization to caveolae in adipocytes [161,166]. In this scenario, a hypothesis for pathological insulin resistance in adipocytes proposes that the dissociation of IR from Caveolin 1 occurs as a result of IR–GM3 interactions in lipid rafts [167]. In fact, this is a plausible mechanism to explain how certain L_o_ membrane microdomains impair insulin signaling. PKC is also modulated by omega-3 PUFAs in diabetic patients and in diabetes, DAG levels are chronically elevated in many peripheral tissues, leading to abnormal PKC activation. In this context, DAG favors the occurrence of L_d_ membrane microdomains which induce the recruitment of PKC to the plasma membrane and its ensuing activation [8,14,88]. Activated PKC enhances IRS (insulin receptor substrate) phosphorylation at Ser/Thr residues, which inhibits a conformational change in IRS that is necessary for IR-mediated Tyr phosphorylation and insulin signaling via PI3K [168]. However, omega-3 PUFAs inhibit PKC to favor insulin signaling [169]. The lipid composition of the plasma membrane also influences glucose transport via GLUT. Indeed, epidemiological studies indicate that dietary changes from unsaturated towards saturated lipids inhibit the insertion of GLUT4 into the plasma membrane, thereby altering glucose uptake from the blood and insulin sensitivity [170]. By contrast, experimental Chol depletion increases the density of GLUT4 receptors at the plasma membrane [171]. Interestingly, GLUT4 translocation to the plasma membrane is, in part, controlled by activation of the IR–IRS–PI3K axis which means that an increase in membrane fluidity (mediated by PUFA enrichment) in the presence of insulin may activate *GLUT4* translocation to the plasma membrane [172]. Finally, *GLUT4* expression is under the control of PPARγ, such that the presence of DHA in cell membranes and an optimal omega-3 to omega-6 ratio may promote GLUT4 expression [169]. Together, this evidence suggests that the membrane lipid composition acts as a switch that regulates the cell’s sensitivity to insulin, whereby lipids that promote membrane fluidity like omega-3 PUFAs potentiate the insulin response and activate the enzymatic machinery for glucose uptake.

There is abundant evidence demonstrating the association between dietary fats and diabetes, which supports the use of dietary fat interventions and melitherapy as therapeutic strategies in diabetic patients. A meta-analysis of studies involving T2DM patients concluded that diets with high MUFA content (33% of the total energy in the form of fat) resulted in lower insulin requirements and decreased glycaemia than low-fat diets (25% of the total energy in the form of fat: [173]). In this context, high oleic acid (OA) intake improves the glycemic status and reduces the saturated FA levels in diabetic patients, while increasing the unsaturated FA content [21]. Moreover, high OA consumption ameliorates the health status of diabetic patients while regulating the lipid content in membranes, which also regulates the membrane association of relevant peripheral proteins [174]. In this scenario, therapy with unsaturated FA derivatives has been shown to reduce glycemia in rats, while other analogues that regulate lipid metabolism prevent T2DM [175]. Therefore, membrane lipid composition is regulated by MUFA intake and that of other fatty acids, controlling insulin sensitivity by modifying the membrane structure [160]. The effect of omega-3 PUFAs in preventing insulin resistance in animals appears to be more robust [176]. A growing body of evidence shows that increases in the unsaturation index in the cell membrane, and particularly in omega-3 PUFAs, is associated with stronger insulin sensitivity [177]. In rats, diets that differ in their FA profile induce marked differences in FA levels in muscle and the liver. Indeed, diets rich in α-linolenic acid or fish oil increase omega-3 PUFAs and lower omega-6 PUFAs [178,179]. In general, improved insulin sensitivity has been associated with the enrichment of omega-3 PUFAs in cell membranes, and although the exact mechanism mediating this effect is not yet fully understood, protein–lipid interactions probably play a relevant role in the control of glycemia [162]. Therefore, the biophysical properties of lipid bilayers and structural membrane dynamics may play crucial roles in diabetic patients that could influence their pathological status and its treatment.

## 8. Protein–Lipid Interactions in Cardiovascular Diseases (CVDs)

The CVDs are the leading causes of death and disability worldwide. They include heart disease, vascular diseases of the brain and other diseases of blood vessels [180]. The major risk factors for CVDs are raised blood pressure (hypertension), raised blood sugar (diabetes) and raised blood Chol (hyperlipidemia), together with other conditions such as cardiac arrhythmia, congenital heart disease, rheumatic heart disease and Chagas disease (American trypanosomiasis).

From the individuals’ perspective, the relevance of lipids in CVD pathogenesis has been widely reported. Raised blood lipids and dyslipidemia promote atherosclerosis and narrowing of the blood vessels. In atherosclerosis, medium- and large-sized blood vessels are subjected to inflammatory processes initiated by the exposure of the endothelium to sustained high levels of low-density lipoprotein Chol (LDL-Chol) or other elements, including free radicals [180,181,182]. The endothelium is then populated by lymphocytes and monocytes that represent the starting point for the formation of atheromatous plaques. A later step is the rupture of the plaque, which produces the release of lipid fragments and cell debris into the lumen of the vessel. In turn, this triggers a cascade leading to thrombus formation which, on reaching a critical size, can block a coronary (in heart attack) or brain vessel (in stroke; [181,182]). Prevention interventions mainly involve the use of aspirin, beta-blockers, angiotensin converting enzyme (ACE) inhibitors and lipid-lowering therapies, together with the cessation of smoking [183].

From the cellular viewpoint, the effects of lipid–protein interactions in CVDs involve lipid rafts (affecting both caveolae and ion channel regulating proteins, among others) and covalent lipid protein modifications. According to their protein and lipid composition, different subtypes of lipid rafts can be distinguished: commonly, caveolae are lipid rafts containing caveolin, while caveolin-free lipid rafts are also found. The two systems where the effects of membrane lipids on CVDs have been studied are endothelial cells of arteries and cardiomyocytes (reviewed in [184,185,186,187]). As for lipid rafts, these are essential elements of endothelial cells involved in vasoconstriction or vasodilation, to which they are associated via the angiotensin II receptor and nitric oxide synthase (eNOS), respectively, consequently affecting hypertension. Nitric oxide (NO) produced by the endothelium promotes muscle relaxation in the smooth muscle cells of the vessel by inhibiting adenylyl cyclase, an enzyme that produces cAMP and that regulates muscle contractility. In cardiac myocytes, lipid rafts are essential for the correct translocation of G proteins that are activated in response to signals initiated by adrenergic and cholinergic receptors. Likewise, the ion channels that control the membrane potential of these cells require lipid rafts for their correct functioning. Furthermore, the channels controlling the cardiac action potential wave require lipid rafts and when their activity is disrupted, life-threatening conditions arise (reviewed in [184,185,186]).

Caveolae are invaginations of the plasma membrane with some lipid raft features and contain caveolins, a family of integral membrane proteins involved in endocytosis [188,189,190]. Some proteins are known to be selectively located in either lipid rafts or caveolae, yet not both [191]. In particular, caveolin-3 must be kept in a very narrow concentration window for the correct regulation of signaling cascades in heart muscle (reviewed in [185]). There is evidence of the involvement of lipid rafts in the pathogenesis of atherosclerosis, especially caveolae (reviewed in [185]), since both the interaction of ApoAI with macrophages and the Chol efflux from these macrophages depend upon lipid rafts and may alter their composition. Data from caveolin-1^−/−^, ApoE^−/−^ and CD36^−/−^ mice further support this assumption [192,193,194].

In terms of covalent lipid modifications, the most obvious one is the need of water-soluble proteins to associate with a lipid moiety in order to interact strongly with membranes or to induce structural changes in these proteins (reviewed in [187]). However, beyond this requirement, lipid peroxidation of unsaturated lipids attacked by oxidants (free radicals, reactive oxygen species—ROS or non-radical species) generate two by-products relevant to CVDs: acrolein and malondialdehyde (MDA). Exposure to substances containing acrolein increases the risk of a CVD. Although the molecular mechanism behind this relationship remains to be fully defined. However, as well as affecting several signaling cascades, in vitro and in vivo studies show alterations to the CV system with a greater tendency towards vasospasm, an increased heart rate, and atherosclerotic lesions and hypertension. As far as MDA is concerned, MDA-modified proteins have been detected in atherosclerotic tissue. Nevertheless, more work is needed to determine the involvement of this molecule in the formation of the atheromatous plaques and in the accompanying molecular changes.

Lipid molecules that alter lipid–protein interactions may have therapeutic value in CVDs. Dietary control is one of the main tools in the prevention of CVD and in therapeutic terms [180], as supported by a significant number of observational and interventional studies [180,195]. The benefits of the Mediterranean diet for CVDs have become generally accepted and recent studies detail the usefulness of dietary supplementation strategies based on this diet. In particular, extra virgin olive oil or mixed nuts decrease the cases of stroke, myocardial infarction and CV mortality [196]. Furthermore, the reduction in the incidence of major CV events is stronger than those due to following a reduced-fat diet [197]. There are several molecular entities that affect lipid–protein interactions and that may underlie these benefits. The levels of specific FA moieties (both in phospholipids and Chol esters) increase upon olive oil consumption and this produces an increase in the MUFA:SFA (saturated FA) ratio. This increase alters membrane lipid structure and membrane fluidity, favoring non-lamellar membrane structures, and affecting the position and activity of certain proteins like G proteins and PKC (reviewed in Reference [198]). Both GPCRs and G proteins are sensitive to the lipid environment [25] and the membrane-association of G proteins (active/pre-active Gαi, Gαo and Gβ) and PKC is significantly impaired in hypertensive subjects. Adrenergic receptors are especially relevant for CVDs, the levels of which vary with age and they can be targeted with beta-blockers. In particular, beta-adrenergic mediated vasorelaxation and Gαs coupling decreases with age and thus, melitherapy seems a plausible strategy to counteract this reduction (reviewed in [198]). The levels of lipoprotein lipase (LPL), a water-soluble enzyme responsible for hydrolyzing triglycerides in lipoproteins, and for the uptake of Chol-rich lipoproteins and of FFAs, decrease upon olive oil supplementation. This change is mediated by microRNA-410, which targets the 3’untranslated region of the *LPL* gene [199].

As previously indicated, hypertension is a major risk factor for CVDs which is accompanied by alterations in membrane Chol or phospholipid content, as well as in the degree of FA saturation and phospholipid distribution [200,201,202]. Indeed, several approaches have been developed to target these molecular alterations. For example, the MUFA 2-hydroxyoleic acid (2OHOA) is a synthetic non-β-oxidation-metabolizable derivative of OA, inspired by the beneficial effects on hypertension of long-term high-dose OA supplementation [203]. The anti-hypertensive potential of 2OHOA was shown in Sprague–Dawley (S–D) and spontaneously hypertensive rats (SHRs), both through intraperitoneal and oral administration [11,204]. Sustained, time-dependent decreases in blood pressure were reported that did not affect heart rate. At the molecular level, there was more Gαs in the aorta and heart membranes of S–D rats, and Gαq/11 and PKCα in heart membranes alone, producing increased cAMP and promoting vasodilatation. Treatment of SHRs with 2-OHOA produced a normalization of the aortic Rho kinase, suppressing the vasoconstrictor Rho kinase pathway seen in SHRs. The prenatal supply of omega-3 fatty acids plays an important role in the development of the CV system and in the regulation of blood pressure [205,206,207]. As such, α-linolenic acid decreases the hypertension derived from omega-3 PUFA deficiency [208], which might be mediated by blood leptin, although this remains to be confirmed.

Another major risk factor of CVDs is raised blood Chol, which has led to the development of a group of drugs used to lower Chol and triglycerides in patients with elevated Chol. Subsequently, a plethora of studies have been carried out on 3-hydroxy-3-methylglutaryl coenzyme A (HMG CoA) reductase inhibitors, the so-called statins (reviewed in [209]). Statins block the Chol synthetic pathway in the liver and indeed, these therapies promote a regression and/or delay in the progression of atheromatous plaques. In addition, hypercholesterolemia induces molecular changes in platelets, altering their membrane phospholipid and Chol composition. These changes make platelets more prone to form aggregates and therefore, statins also reduce the formation of blood clots. However, the precise mechanism underlying the effects of statins on platelet function remain uncertain and could involve reduced TXA2 production in platelets (reviewed in [210]). An alternative agent to lowering Chol is dextrin, which depletes membrane Chol and compromises caveolae stability. It has been reported that cyclodextrin impairs adenylyl cyclase function (reviewed in Reference [185]) and more recently, in vivo studies on SHRs showed this drug to induce Serine1177 phosphorylation of eNOS and increased ROS production [211].

Finally, raised blood Chol is also a major risk factor for CVDs. The clinical benefits of statins in CVDs has been described (reviewed in [210]), although the molecular mechanisms remain to be determined. Alternatively, the contribution of altered lipid profiles to damage following stroke was proposed almost 25 years ago (reviewed in [212]). Stroke-induced energy failure is followed by FFA release from the plasma membrane of damaged cells, some of which expand ischemic damage (for example, omega-6 AA), while others exert a pro-survival effect. AA is subject to the action of cyclooxygenases (COX) and lipoxygenases (LOX), converting it into metabolites that act as proinflammatory eicosanoids (prostaglandins, thromboxanes and leukotrienes). Accordingly, 2-hydroxy arachidonic acid (2-OAA) is a rationally designed derivative of AA known to be a competitive inhibitor of COX-1 and COX-2, and thus, it can be used in LPS-treated mice to decrease proinflammatory cytokines in serum (reviewed in [212]). When assessed for the treatment of stroke using S-D rats, 2-OAA treatment in the first hour of reperfusion after the stroke produced a neuroprotection [213]. At the molecular level, 2-OAA decreased phospholipase A2 (PLA2) in the cell membrane with a subsequent decrease in FFA release. A decrease in oxidative stress also occurs upon 2-OAA treatment in the first hour of reperfusion after stroke. Thus, the use of rationally designed lipids would seem to be a promising new stroke therapy, with an important advantage that they cross the Blood–Brain Barrier [213].

## 9. Protein–Lipid Interactions in Infectious Diseases

Bacterial membranes show important differences with respect to eukaryotic cell membranes [214], which has two relevant implications: first, different types of protein–lipid interactions can be seen; and second, these differences may permit the development of new therapeutic strategies to treat infectious diseases, using compounds that produce specifically affect only on prokaryotic cell membranes. Given the increased resistance of infectious microorganisms to conventional antibiotics, antimicrobial peptides are potentially interesting treatments to combat infections. As such, several antimicrobial peptides with potential therapeutic activity have been found in the skin of amphibians, such as magainin 2, bombinin, caerin 1.1, etc. [215,216,217]. The specific or preferential interactions of these peptides with bacterial membranes can induce relevant alterations in the lipid bilayer, such as the thinning of the lipid bilayer or the generation of membrane pores, causing the release of intracellular content or the uncoupling of respiration in bacteria [215,216,217]. Many natural or designed antimicrobial peptides have been described that interact distinctly with eukaryotic membranes, involving electrostatic or hydrophobic interactions, specific interactions with certain membrane lipids, intercalation of the interfacial region of polar heads or within the hydrophobic membrane core, etc. Indeed, a database containing currently known antimicrobial peptides has become available [218].

There are two further examples supporting the relevance of protein–lipid interactions in infectious microorganisms [219,220], in which the selectivity of lipid binding to membrane protein complexes has been explored. In the first, a mass spectrometry (MS) study of three different membrane protein complexes aimed to examine several topologies, oligomeric states and selectivity for lipids [219]. The second study described the direct protein–lipid interactions that shape the conformational landscape of secondary transporters, using hydrogen–deuterium exchange mass spectrometry (HDX-MS) [220]. In this latter study, modeling was performed using the major facilitator superfamily (MFS), which includes thousands of closely related secondary active and passive solute transporters, such as multidrug efflux pumps [221,222,223]. Most of these proteins are 500 residue long single polypeptide chains with 12 to 14 transmembrane segments (TMS). The MFS group includes most of the known secondary transporters, such as transporters implicated in many human pathologies, in resistance to chemotherapeutic agents in humans and in resistance to antibiotics in bacteria (reviewed in [224,225]). Direct interactions between PE and the charge networks stabilize the inward-facing conformation, facilitating substrate release into the cytosol. It was therefore speculated that conformational regulation by specific lipid–protein interactions constitutes a widespread mechanism employed by many transporters, such as the clinically relevant solute carrier (SLC) transporters [226]. Both these studies illustrate how lipids fine tune the structure and function of membrane proteins, through their relative abundance and the differences in their selectivity for amino acid residues [227]. Specifically, in infectious diseases this regulation influences both the interaction of the pathogenic organism with the host cell and the reaction of the immunological cells involved in the response to the pathogenic organism or condition. Table 1 summarizes several examples of lipid structures involved in various pathological conditions affecting the immune system.

## 10. Protein–Lipid Interactions and Cell Switches

A cell is a complex compartmentalized structure with organelles bounded by membranes made up of one or two lipid bilayers. Many events occur in the aqueous fraction of these organelles, whereas other functions take place within or around their membranes. In fact, most cell functions take place in or around membranes, highlighting the relevance of these structures. A classic example of this environmental control of protein function is the regulation by protein–membrane interactions [98]. As the close proximity of soluble proteins to various lipids in the cytoplasm is inevitable, soluble proteins often interact with the membranes of different intracellular organelles and vesicles. We have investigated the interaction of multiple proteins with membranes and we found that many pathophysiological situations involve altered protein–lipid interactions. Moreover, important changes in the cell’s physiology are associated with dramatic changes in the membrane lipid composition, which drives important variations in cell signaling. With evolution, these interactions became regulatory switches that control the activity of one or more proteins, modulating their shift from the cytosolic to membrane fractions [231]. For other proteins, such as those in the cytoplasm or serum [232], membranes can provide a general stabilizing micro-environment. For example, the interactions of the glycogen branching enzyme [26], nitroreductase [233], and brain spectrin [234] with model membranes demonstrate that these soluble proteins can be regulated by lipid bilayers.. Here we will describe some of the main cellular processes controlled by the reversible interaction of proteins with intracellular membranes and lipids.

To serve as mediators of cell responses, the activity of peripheral proteins must be dictated by spatiotemporal-specific interactions with signaling lipids. This is indeed the case for many functional protein–lipid interactions, such as Akt [235]. Thus, Akt is a master activator of cell anabolism, promoting cell growth by stimulating cell cycle progression [236], as well as insulin-dependent glucose uptake and biosynthesis [237,238,239] while inhibiting apoptosis [240,241] and transcription factor EB (TFEB)-dependent degradative lysosomal/autophagosomal pathways [242]. All these coordinated cell growths promoting activities are switched on or off by Akt binding to, and dissociating from, signaling lipids. For example, Akt is translocated from the cytosol to the plasma membrane (switched on) by binding to PtdIns(3,4,5)P3—a lipid formed by PI3 Kinase (PI3K)-mediated phosphorylation of PtdIns(4,5)P2. Interestingly, PI3K is mainly activated by receptor tyrosine kinases (RTKs) or GPCRs as a result of distinct extracellular anabolic cues, such as insulin, growth factors, cytokines and hormones. Alternatively, the tumor suppressor phosphatase and tensin homologue (PTEN) can switch off Akt growth promotion by dephosphorylating PtdIns(3,4,5)P3 to PtdIns(4,5)P2, translocating Akt from the plasma membrane back to the cytosol and terminating its downstream signaling.

However, not all protein–membrane lipid interactions involve signaling lipids. In general, proteins like Akt that interact with signaling lipids have a high basal affinity for membranes containing those lipids, enabling them to quickly respond to short-term signaling lipids. By contrast, proteins interacting with bulk lipids usually have a low basal affinity for membranes containing those lipids, and their membrane translocation from the cytosol is triggered by electrostatic (e.g., Ca^2+^ binding) and conformational (e.g., surface exposure of hydrophobic patches) modifications, or phosphorylation [243]. One of the best studied examples of proteins interacting with bulk membrane lipids is PKCα, which contains a conserved C2 domain that upon Ca^2+^ binding, is selectively translocated to PS-rich plasma membrane microdomains, although Ca^2+^-activated C2 can also bind PC. This selectivity for PS over PC is provided by an additional structural buttress to the C2 domain, which is PKCα-Asn189. Moreover, DAG and PE also participate in the interaction of various PKC isozymes (see above). The PC selectivity of the nuclear membrane is due to another bulk lipid interacting protein, cytosolic pholspholipase A2 (cPLA2), which is produced by Ca^2+^-dependent exposure of hydrophobic and aromatic residues [244].

**Figure 5:**
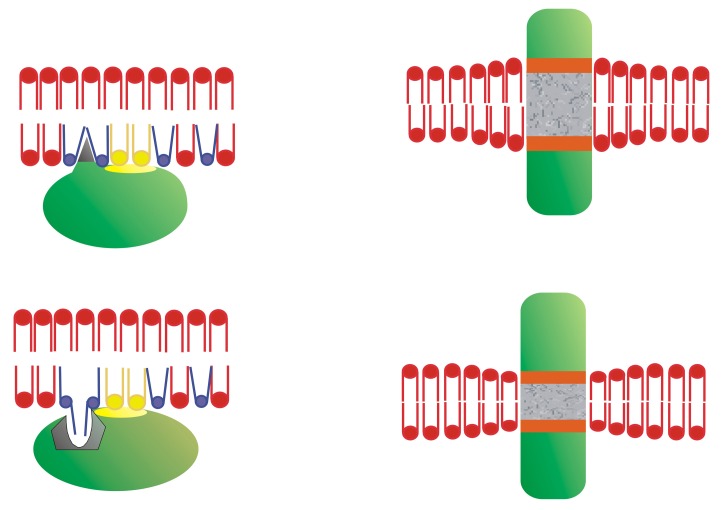
Membrane lipid structure and protein interactions. (**Left**) The interaction of amphitropic peripheral proteins with non-lamellar-prone bilayers with loose surface packing. (**Right**) The Interaction of integral transmembrane-spanning proteins with lipid bilayers. Adapted from Reference [20].

The mechanism by which membrane lipid–protein interactions activate downstream pathways is based on the regulation of membrane lipid structure and composition, altering the localization and activity of signaling proteins. This constitutes the basis of the innovative melitherapy approach [98]. For instance, an increase in the non-lamellar phase propensity of membranes induced by synthetic lipids like 2OHOA [21,98,245] can enhance PKC binding to these membranes [246]. This increased non-lamellar-phase propensity reduces the lateral surface pressure of the lipid bilayer, which enables hydrophobic domains of other peripheral proteins to interact with deep hydrophobic regions of the membrane and/or fatty acid moieties of phospholipids that protrude out of the bilayer plane (Figure 5). Interestingly, swelling-induced mechanical stretching of the nuclear membrane allows it to accommodate the amphitropic cytosolic PLA2 protein [247]. Changes in membrane composition may also cause selective membrane lipid anchoring. Thus, increasing the levels of DAG by 2OHOA mediated transfer of the polar head of PC to ceramide can also enhance PKC binding to membranes through its interaction with DAG [248]. Changes in lipid composition induced by 2OHOA also cause Ras depletion from the plasma membrane and the attenuation of its downstream oncogenic signaling [21,88]. Indeed, the synthetic lipid hydroxytriolein, which like 2OHOA also regulates membrane lipid composition, and causes cytotoxicity in triple negative breast cancer cells [249]. One of the pathways affected augments the ceramide and acyl glycerol in membranes, which can recruit PKC to them, and initiate differentiation and growth arrest responses through PKC-dependent signaling cascades.

A number of cellular activities can be induced by lipid–protein interactions, such as proliferation. One of the mechanisms that normally regulates the transition of cells from a quiescent to proliferative state (and vice versa) is the so-called proliferation switch [98]. The transfer of proteins to and from intracellular membranes can initiate signals that determine cell fate (proliferation, quiescence or programmed death). In many tumors, the membrane lipid composition permits the continuous and intense interaction of proliferative proteins that propagate cell growth [21,40,250]. Membranes undergo relevant lipid changes between the quiescent and proliferative state that modulate protein sorting to cell membranes and the signals they drive. Altering this membrane lipid switch (i.e., changing the membrane lipid composition) may affect the localization and activity of proteins that promote proliferation, such as the farnesylated small GTPase Ras [251,252], and it also regulates membrane anchoring of tumor suppressors like PKC. Certain synthetic lipids like 2OHOA [88] and hydroxytriolein [249,253] alter the membrane’s composition, reversing cancer cell proliferation and preventing the uncontrolled growth of cancer cells by relocating Ras to the cytosol [21], FoxO1 to the nucleus, PKC to the plasma membrane [21,98,245], or PKC to the cytosol through hydroxytriolein activity [253]. These alterations attenuate cancer cell proliferation, and induce differentiation, ER stress and ERK-dependent autophagic cell death.

A second activity regulated by membrane lipid switches is that of enzymes involved in lipid metabolism. Phospholipases hydrolyze phospholipids into FAs and other products, and they are classified according to their phospholipid cleavage site. The cytosolic PLA2 family (cPLA2), or the Group IV cPLA2, are six enzymes that hydrolyze phospholipids at the sn-2 site to generate the AA and lysophosphatidic acid (LPA) signaling molecules. Thus, cPLA2 has been studied intensely and interestingly, cPLA2 is reversibly translocated to membranes in a stimulus-dependent fashion, usually in response to receptor activation. The cPLA2 enzymes are recruited to membranes by direct binding to their phospholipid substrates, yet they can only hydrolyze their substrates when allosterically activated at their interfacial surface at the membrane, rather than being regulated by their substrate [254]. Membrane binding of both cPLA2 and PKC is mediated by their negatively charged C2 domain, which binds to positively charged Ca^2+^ ions released upon stimulation, becoming more hydrophobic (less charged: [255]). The enzyme, PKC, usually binds to PS (and PtdIns and DAG) in the plasma membrane, while cPLA2 binds to PC in intracellular membranes, the specificity of which is determined by the Ca^2+^ levels [244,256,257]. In addition, PLCβ phospholipases also offer another interesting level of control of phospholipase membrane translocation. An elegant total internal reflection fluorescence study demonstrated that PLCβ1a is dissociated from the plasma membrane upon activation by agonist binding to a GPCR which, in turn, activates a Gαq protein [258]. However, PLCβ1a dissociation from the membrane is not mediated by Gαq interactions but is rather caused by GPCR activated dephosphorylation of the PLCβ1a substrate PtdIns(4,5)P2.

A third type of activity regulated by lipid switches is that catalyzed by lipid transport proteins. Non-vesicular lipid transport (NVLT; reviewed in detail in [259]) involves highly specific interactions between lipids and their intracellular carriers, involving hundreds of different lipid transfer proteins (LTPs) [259]. This NVLT has been studied extensively over the past two decades and it is now known that most lipids reach their destinations by non-vesicular rather than vesicular transport. This conclusion was drawn from the high rate of inter-organelle phospholipid and Chol transport, which could not have been obtained by vesicular transport [260,261,262], as well as from studies in which chemical and genetic interference of the secretory pathway left lipid transport from the ER to the plasma membrane [261,263,264]. Therefore, NVLT is important because it is the only route to supply lipids to organelles that do not partake in vesicular transport within the cell, such as mitochondria and peroxisomes. Moreover, as opposed to vesicular transport that can only offer the unsorted bulk supply of lipids, including membrane proteins, NVLT permits the regulation of membrane lipid composition. Finally, NVLT permits metabolic control. For example, ceramides transported from the endoplasmic reticulum (ER) to the Golgi complex can only be converted into glycolipids and sphingolipids if they are transported selectively. The steroidogenic acute regulatory protein (StAR) that transports Chol from the outer to the inner mitochondrial membrane for steroid synthesis in the matrix is another example of this [265]. Lastly, the generation of cardiolipin and PE by enzymes of the inner mitochondrial membranes that use PA and PS as substrates delivered from the ER [266] is also a unique metabolic pathway. This pathway is the only lipid biosynthetic reaction not confined to the ER and cardiolipin is one of its products unique to mitochondria. NVLT also allows lipids to be used in signaling. For instance, the selective transport of DAG by E-Syt to membranes containing PKC [259], or the selective transport of ceramides by CERT to mitochondria rather than the Golgi complex [267], can both switch programmed cell death. In the context of signaling, it is also important to note that many LBPs can act as lipid chaperones and sensors: FABP5 chaperones retinoic acid to the nuclear PPAR-δ receptor, activating the transcription of several target genes [265]. Alternatively, the oxysterol binding protein (OSBP) binds Chol when its intracellular concentrations rise, provoking the attenuation of the ERK pathway [265].

The plasma membrane can sense changes in temperature and other cellular stresses. Such changes can provoke a reorganization of the membrane, which in turn modifies the interaction of different receptors and proteins that control cellular stress, including the heat shock response (HSR: [268]; reviewed in [21,269,270]). The modulation of lipid microdomains in membranes in response to environmental stimuli induces the expression of Heat shock proteins (Hsps: [268]), which act as chaperones to resolve protein misfolding [271]. Some of these chaperones localize to the extracellular space in cancer and other pathological situations, such as Hsp70 and Hsp72 [272,273], or to glycosphingolipid and Chol rich microdomains (GCMs: [274,275]). Indeed, Hsp70 can bind to the plasma membrane through the Gb3 present in GCMs, a phenomenon that can be reverted by Chol depletion [276]. Moreover, Hsp70 and other chaperones modulate their localization and lipid interactions during stress, which regulates lysosomal homeostasis as well as endocytosis, apoptosis and survival signaling [270].

In the same context, the modification of the plasma membrane can regulate the activity of HSF1 (heat shock transcription factor 1), without inducing cellular stress, and this protein is responsible for inducing chaperone expression by binding to heat shock promoter elements, (reviewed in [264]). The changes in the membrane modulate the activity of the TRPV channels, altering the Ca^2+^ available in the cell, which activates IP3 and DAG signaling, as well as the PKA and MAPK signaling pathways, and modifies growth factor receptor (GFR) activity [269]. Moreover, the use of the membrane fluidizers benzyl alcohol (BA) and heptanol in model membranes [277,278] as well as the addition of cholesteryl glucoside to TIG3 cells [279], mimics the Hsp response and the HSR triggered by thermal stress. The lipid bilayer structure also acts as a thermal sensor for temperature changes (e.g., the heat shock response), which highlights its potential as a therapeutic target in a number of pathologies. Lipid structure changes due to thermal changes induce HSR via Hsp/HSF activation, and thus HSR can be considered a “lipid switch-induced” response. Thus, this strategy has been used for the investigation of membrane lipid fluidizers, which can activate the HSR, as medicinal drugs for the treatment of human diseases (e.g., BGP-15) [280].

In controlling cellular stress, there are some lipids that can act as non-protein molecular chaperones, such as PE [281,282,283]. PE with two saturated fatty acids and Lyso-PS can refold a lipid-dependent epitope of lactose permease in vitro, while PC and PE with unsaturated fatty acid chains were ineffective in this regard [282]. Similar activity has also been reported in the case of heme proteins like horseradish peroxidase (HRP) [283]. In addition to their role as chaperones, the γ-ketoaldehydes that result from lipid peroxidation modify the amino group in PEs and they alter membrane curvature and ER stress markers, such as CHOP and BiP, as well as endothelial activation in human umbilical cord endothelial cells [284]. Moreover, lysophospholipids in *Escherichia coli*, mainly in the form of lysophosphatidylethanolamine, display chaperone-like activity, preventing the aggregation of citrate synthase in heat shock conditions (42 °C: [285]).

A further example of general (switch) regulatory process is the regulation of caveolae function. Caveolae are inward facing bulb-shaped invaginations of the plasma membrane and they fulfil multiple functions in signal transduction, mechanoprotection and endocytosis. The implication of integral and peripheral membrane proteins and lipids in the activity of caveolae indicate that they are controlled by various lipid switches. Caveolae only form at the cytosolic face of the plasma membrane, further suggesting that lipid composition and unique lipid–protein interactions are core aspects of caveolae regulation. Indeed, cavins are peripheral proteins that form caveolae by interacting with integral caveolin proteins in the membrane, and which have PS and PI(4,5)P2 binding sites [286]. However, cavin recruitment to caveolae is also dependent on the binding of caveolin CAV-1, indicating that lipid switches may also involve multiple low affinity interactions of both lipids and proteins. Interestingly, the PI(4,5)P2-binding site in cavin1 is mutually exclusive with ubiquitin binding, which leads to proteasomal degradation [286]. Thus, under conditions of mechanical stress at the plasma membrane, such as osmotic swelling, less cavin1 is released from the plasma membrane and an equilibrium relative to the levels of CAV1 at the plasma membrane persists. Hence, caveolae dynamics (generation and degradation) are maintained through protein–lipid interactions. This observation is further corroborated by a study which showed that the budding of caveolae in CNS endothelial cells at the blood-brain barrier and the ensuing transcytosis is inhibited by PUFA containing phospholipids [287], again demonstrating how caveolae function is switched on and off by specific lipid–protein interactions.

In conclusion, we showed here that the reversible interaction of proteins with specific membrane structures or membrane lipids can switch various cellular functions on or off, or even alter general cellular processes. These activities range from cell fate determination (cell growth, differentiation or death), to intracellular trafficking through specific enzyme reactions and chaperone activities. This situates lipid–protein interactions as important targets for pharmacological intervention, which can reverse detrimental cell responses and provide remedies to a variety of diseases.

## Figures and Tables

**Table 1:** Lipid structures involved in various pathological conditions affecting the immune system.

Lipid Element	Protein Element	Pathogenic/Physiological Condition	Lipids Implicated in Pathogenicity	Therapeutic Approach Targeting the Lipid Fraction	Reference
Lipid rafts PUFAs	IL-2, FcR, PKC, NF-kB, AP-1	Altered localization of receptors, mediators and transcription factors	PUFAs	Dietary supply of PUFAs alters T- and B-lymphocyte membranes	[185]
Lipid rafts PUFAs	PTKs (LCK), CD45, CD3, FcR	SLE	Increased amount of lipid rafts in activated T-cells	-	[185]
PE	Atg8/LC3	Double membrane formation of the autophagosome	-	-	[129]
Palmitoyl moeity	TLRs	Innate immune response, regulation of immune receptor functions	-	-	[129,228]
Several lipid moieties	Several proteins	*Plasmodium falciparum* (malaria)	-	NMT validated as an attractive antimalarial drug target	[129]
Several lipid moieties	Several proteins	*Trypanosoma brucei* (human African trypanosomiasis)	-	NMT identified as a promising target for sleeping sickness (inhibitor DDD85646)	[129]
Fatty acylation	Rho-family GTPases (lysine residues)	*Vibrio cholera*	Toxin peptide catalyzing the fatty acylation of lysine residues of Rho-family GTPases	-	[229]
Chol	CR3 and others	*Mycobacterium tuberculosis*	Extractable lipids they are important virulence factors	Host Chol is required for receptor-mediated phagocytosis of *M. tuberculosis* by a macrophage. Blocking antibodies showed that Chol is required for mycobacterial entry via CR3. Statins showed promise in vitro and in vivo for the treatment of tuberculosis	[230]
Diverse lipid moieties	Several proteins	Herpes simplex virus	-	-	[129]
Lipid rafts	CD4	HIV infection	PUFAs, increased amount of lipid rafts	Disruption of host cell lipid rafts with cyclodextrin prevents HIV infection. Inhibiting sphingolipid synthesis by the virus particle reduces its infective capacity.	[185]
Myristoylation	Gag protein	HIV infection		Targeting lipidated viral or host proteins may lead to new antiviral agents.	[129,230]
Chol	Gp41 fusion protein	HIV infection	-	-	[129,230]
Phosphoinositides	-	HIV infection	Effect on positive membrane curvature	-	[230]
Lipid rafts, edges of Chol-rich domains	CD4-CCR5/CXCR4	HIV infection	Effect on the budding out of the host cell	-	[230]
Diverse lipid components	Gag-Gag, GPCR	HIV infection	Effect on the budding out of the host cell	-	[230]
Diverse lipid components	Gag multimerization	HIV infection	Budding virus are enriched in several lipids compared to the plasma membrane composition of the infected cells from which they originate	-	[230]

Abbreviations: AP-1, activator protein 1; Atg8, autophagy-related protein 8; CCR5, C-C chemokine receptor type 5; CD3, cluster of differentiation 3; CD4, cluster of differentiation 4; CD45, cluster of differentiation 45; CR3, complement receptor 3; CXCR4, C-X-C chemokine receptor type 4; FcR, Fc receptor; IL-2, interleukin 2; LC3, light chain 3; LCK, lymphocyte-specific protein tyrosine kinase; NF-kB, nuclear factor kB; NMT, N-myristoyltransferase; PKC, protein kinase C; PTKs, tyrosine-protein kinase; SLE, Systemic lupus erythematosus; TLR, Toll-like receptors; HIV, Human Immunodeficiency Virus.

## Abbreviations

2OAA	2-hydroxy arachidonic acid
2OHOA	2-hydroxyoleic acid
AA	Arachidonic acid
ACE	Angiotensin converting enzyme
AD	Alzheimer’s disease
APP	Amyloid precursor protein
BA	Benzyl alcohol
Chol	Cholesterol
CLPs	Covalent-lipid proteins
CNS	Central nervous system
COX	Cyclooxygenase
cPLA2	Cytosolic phospholipase A2
CVDs	Cardiovascular diseases
DAG	Diacylglycerol
DHA	Docosahexaenoic acid
EGF	Endothelial growth factor
eNOS	Nitric oxide synthase
ER	Endoplasmic reticulum
FABPs	Fatty acid binding proteins
FFAs	Free fatty acids
FTase	Farnesyl transferase
GCMs	Chol-rich microdomains
GFP	Green fluorescent protein
GFR	Growth factor receptor
GGTase	Geranylgeranyl transferase
GPCRs	G-protein coupled receptors
GPR40	G-protein coupled receptor 40
HDX-MS	Hydrogen–deuterium exchange mass spectrometry
HII	Non-lamellar-prone
HMG CoA	3-hydroxy-3-methylglutaryl coenzyme A
HRP	Horseradish peroxidase
Hsp	Heat shock protein
HSR	Heat shock response
ICMT	Isoprenyl carboxyl methyltransferase
IR	Insulin receptor
IRS	Insulin receptor substrate
LBPs	Lipid-binding proteins
Ld	Liquid disordered
LDL-Chol	Low-density lipoprotein Chol
Lo	Liquid ordered
LOX	Lipoxygenase
LPA	Lysophosphatidic acid
LPC	lysoPC
LPE	lysoPE
LPL	Lipoprotein lipase
LPS	LysoPC
MBP	Myelin basic protein
MDA	Malondialdehyde
MFS	Major facilitator superfamily
MLT	Membrane-lipid therapy, melitherapy
MS	Multiple sclerosis
MS	Mass spectrometry
MUFAs	Monounsaturated fatty acids
NO	Nitric oxide
NPD1	Neuroprotetin D1
NSPCs	Neural Stem/Progenitor Cells
NVLT	Non-vesicular lipid transport
OA	Oleic acid
OSBP	Oxysterol binding protein
PC	Phosphatidylcholine
PD	Parkinson’s disease
PDK1	Phosphoinositide-dependent kinase 1
PE	Phosphatidylethanolamine
PH	Pleckstrin homology
PI3K	PI3 Kinase
PKC	Protein kinase C
PLA2	Phospholipase A2
PLCβ	Phospholipase Cβ
PPAR	Peroxisome proliferator-activated receptor
PS	Phosphatidylserine
PtdIns	Phosphoinositides
PTEN	Phosphatase and tensin homologue
RCE1	Ras converting enzyme 1
RTKs	Receptor tyrosine kinases
SAMs	Sterile alpha motifs
S–D	Sprague–Dawley
SFA	Saturated fatty acid
SHRs	Spontaneously hypertensive rats
SLC	Solute carrier
SM	Sphingomyelin
StAR	Steroidogenic acute regulatory protein
T2DM	Type-2 diabetes mellitus
TFEB	Transcription factor EB
TMS	Transmembrane segments
VEGF	Vascular endothelial growth factor
